# Interplay among positive and negative symptoms, neurocognition, social cognition, and functioning in clinically stable patients with schizophrenia: a network analysis

**DOI:** 10.12688/f1000research.74385.2

**Published:** 2022-03-11

**Authors:** Thammanard Charernboon

**Affiliations:** 1 Faculty of Medicine, Thammasat University, Pathumthani, 12120, Thailand; 2 Center of Excellence in Applied Epidemiology, Thammasat University, Pathumthani, 12120, Thailand

**Keywords:** Negative symptoms, network analysis, neurocognition, schizophrenia, social cognition

## Abstract

**Background:** Schizophrenia has a broad range of interrelated symptoms and impairment in functioning. The objective of the study was to explore the interplay between positive symptoms, negative symptoms, neurocognition, social cognition and functional outcome in patients with schizophrenia using network analysis.

**Methods:** Participants were 64 clinically stable patients with schizophrenia. Psychopathologic, neurocognition, social cognition, and functional outcome were measured using the Scale for the Assessment of Positive Symptoms, Scale for the Assessment of Negative Symptoms, Addenbrooke’s Cognitive Examination III, Faces test, Reading the Mind in the Eyes test, and Personal Social Performance scale.

**Results:** The network analysis suggested that functional outcome was the most central in the network followed by avolition and asociality. Functioning was directly connected to avolition, asociality, blunted affect, neurocognition and emotion recognition. The positive symptoms were the most remote and therefore the least important node.

**Conclusion**: The high centrality of functioning suggests the need for improving of everyday life skills for patients with schizophrenia. Moreover, treatment of specific negative symptoms, neurocognition and emotion recognition could also enhance functional outcome.

## Introduction

Schizophrenia is associated with a broad range of symptoms including positive symptoms, negative symptoms and neurocognition impairments. Recently, social cognition deficits also have been increasingly reported. ^1^^,^^2^ Despite the increase in new medications and treatments, functional outcome is not as good as expected. Functional recovery is observed in less than 25% of patients with schizophrenia. ^3^

While all of the symptoms are believed to affect functional outcome to some extent, negative symptoms, neurocognitive, and social cognition seem to have the highest impact on functioning. ^4^ For example, previous studies generally demonstrate that negative symptoms have associations with poorer levels of functioning. ^5^ Neurocognitive impairment is a predictor for low levels of a real-world functioning. ^6^ Moreover, in the last decade, social cognition, a relatively new concept, appears to be a strong predictor of social abilities and real-world function. Theory of mind and emotion recognition are the most extensively studied domains in social cognition study, and impairment in these tasks are risk factors for poor social and functional outcome in patients with schizophrenia. ^4^^,^^6^

However, the interplay between these symptoms and functioning is highly complex. Since not only can negative symptoms, neurocognition, and social cognition predict functional outcome, all of these symptoms are also interrelated to each other. For example, previous studies demonstrated that negative symptoms were closely associated with social cognition. ^7^^,^^8^ Social cognition and neurocognition are also usually correlated to some degree. ^9^ Moreover, some studies suggest that social cognition might be a mediator between neurocognition and symptomatology. ^10^ Therefore, as noted before, a traditional statistical approach might not be suitable for examining complex relationships of interconnected variables. For instance, a simple correlation approach could not control for the influence of other variables. Linear regression and structural equation models require a priori assumptions regarding the selection of predictors, mediators, precursors, and outcomes. Furthermore, traditional analysis could not demonstrate which variables connect to other variable more often and which ones play a more or less central role than others.

Network analysis is a relatively new and powerful methodological approach to investigate complex relationship patterns. It is a data-driven technique that does not require a priori assumption of relationships among variables. With a network analysis approach, all phenomena are conceptualized as systems of causally connected signs and symptoms. The system can be analysed and presented in its full complexity. ^11^ In the network, the key variables will be located at the center of the network, while less important and connected variables will be in the periphery. The aim of this study was to explore the interplay of positive symptoms, five groups of negative symptoms, neurocognition, two forms of social cognition (theory of mind, emotion recognition), and functioning in patients with clinically stable schizophrenia using network analysis.

## Methods

### Participants

64 outpatients with schizophrenia were recruited from a mental health clinic at Thammasat University Hospital, Thailand between February 2018 and August 2019. They were between 20 and 60 years old and had at least four years of education. All patients met criteria for schizophrenia based upon the Diagnostic and Statistical Manual of Mental Disorders-5 (DSM-5) criteria ^12^ and were in a clinically stable phase, defined as no changes in treatment or symptoms for the previous three months. Exclusion criteria were having a major neurological disorder or other psychiatric disorders (i.e., intellectual disability, active major depressive disorder and substance dependence excluding smoking). The participants were invited to participate in this study by our research assistants during outpatient visits. The study was approved by the Human Research Ethics Committee of Thammasat University (No. MTU-EC-PS-0-191/60). Written informed consent forms were obtained from all participants. Participants had a right to withdraw from the study at any time.

### Measures

Positive symptoms

Positive symptoms were measured using the Scale for the Assessment of Positive Symptoms (SAPS). ^13^^,^^14^ Positive symptoms were presented as a single unitary construct using the sum of global ratings. The global ratings range from 0 to 20 with the higher score indicating more severe positive symptoms.

Negative symptoms

The Scale for the Assessment of Negative Symptoms (SANS) was administered to assess negative symptoms. ^15^^,^^16^ In this study, negative symptoms were classified into five subdomains i.e., blunted affect, alogia, anhedonia, avolition, and asociality as suggested by the NIMH-MATRICS consensus statement on negative symptoms. ^17^ Each subdomain has a maximum score of five, which is computed from the average scores of the relevant items of each subdomain. It should be noted that inattention subdomain was not included in the analysis because of its overlap with neurocognitive assessment.

We used five dimensions of negative symptoms instead of a unidimensional approach because several recent factor analysis studies suggest that a unidimensional model of negative symptoms does not adequately capture the complexity of negative symptoms. ^17^^,^^18^ Furthermore, previous study showed that negative symptoms subdomains seem to have a different relationship with functioning. For example, avolition and apathy seem to be a stronger predictor of poor outcomes than blunted affect. ^4^^,^^19^ Therefore, utilizing a multidimensional model would avoid losing information relevant to the connection with other variables and the ability of each subdomain to predict functioning.

Neurocognition

The Addenbrooke’s Cognitive Examination III (ACE-III) was used to assess neurocognitive function. It assesses five neurocognitive domains: attention, verbal fluency, memory, language, and visuospatial ability with a maximum score of 100 points. ^20^^,^^21^A previous study demonstrated that ACE-III was sensitive to detect cognitive impairment in patients with schizophrenia. ^22^ A Thai version of the ACE-III had an excellent inter-rater reliability with a Pearson’s correlation of 1.0 (p < 0.001). ^20^

Social cognition

The social cognition assessments included tests of emotion recognition (Faces test), ^23^ and theory of mind (Reading the Mind in the Eyes test: RMET). ^24^^,^^25^ The Faces test consists of 20 photographs of people faces showing a variety of emotions. The participants were required to match emotions to faces. The maximum score is 20 with a higher score suggesting better emotion recognition ability. ^23^ The RMET includes 36 pictures of persons’ eye regions where participants must select which of four words best describes the mental state of a target person. The RMET has a score range of 0-36 with a higher score indicating better theory of mind capability. ^24^^,^^25^

Real-life functioning

The functioning of the participants was measured using the Personal Social Performance scale (PSP). ^26^ It assessed the patients’ functioning based on four dimensions: useful activities, social relationships, self-care and disturbing/aggressive behaviors. The score ranges from 1 to 100. A score of 91-100 indicates excellent function, while 1-10 suggests lack of autonomy in basic function. ^26^ The Thai version of the PSP had an acceptable inter-rater reliability with the intraclass correlation coefficients of 0.75 (p < 0.001). ^27^

### Procedure

Characteristics and clinical data were retrieved from medical records. Psychiatrists interviewed the patients and rated the SAPS, SANS and PSP. Then, the ACE-III, Faces, and RMET tests were administered by independent clinical psychologists on the same day. All measures were paper versions.

### Statistical analysis

Descriptive statistic and partial correlation were analyzed using STATA version 14.0. Network analysis was conducted using R version 4.0.5 (R Foundation for Statistical Computing). 10 variables were selected for the network analysis. The least absolute shrinkage and selection operator network (LASSO) was used as type of network. The centrality measures of the network were also analyzed. The network was visualized using qgraph package.

A network was created comprising variables, which are represented by ‘nodes’ (circle), and the links between the nodes called ‘edges’ (solid line). Thicker edges represent stronger relationships. Blue edges represent positive correlation, and red edges represent negative correlations. The algorithm places strongly associated nodes at the center of the network and weakly associated variables at the periphery.

The centrality indices are the method to examine the relative centrality of constructs within the network. They reveal which is the most important variable in the network. The common centrality measures are ‘strength’, ‘betweenness’ and ‘closeness’. Node strength reflects how strongly a node is directly connected to other nodes. It was determined by the sum weighted associations to other nodes. Betweenness of a node is defined as the number of times that a node is part of the shortest path between two other nodes. The closeness of the node implies how easy it is to reach all other nodes. A high closeness index indicated a short average distance from an interest node to all other nodes. For each measure, higher values indicated more centrality in the network.

The accuracy and stability of the centrality indices of the estimated network were examined using edge weight accuracy and case-dropping bootstrap method on the 500 samples. We utilized recommended cut-off for Coefficient Stability (CS-coefficient, correlation = 0.7) at least 0.25 for considering stable network. ^28^

## Results

64 patients with schizophrenia were recruited into the study. Characteristics and descriptive statistics of the network analysis variables are reported in Table 1. The patients had a mean age of 37 (standard deviation (SD) 12.6) years and level of education of 13.3 (SD 3.4) years. The average duration of illness was 8 (SD 9) years. Table 2 presents the partial correlation matrix between 10 network analysis variables. There was no missing data in all variables.

**Table 1.** Characteristics and descriptive statistics of variables used in the network analysis.

Variables	Participants (N = 64) Mean (SD)
Gender, male: N (%)	27.0 (42.2%)
Age (years)	37.0 (12.6)
Educational level (years)	13.3 (3.4)
Duration of illness (years)	8.0 (9.0)
SANS-blunted affect score	0.8 (0.8)
SANS-alogia score	0.6 (0.6)
SANS-avolition score	1.2 (1.0)
SANS-anhedonia score	1.1 (1.2)
SANS-asociality score	2.0 (1.2)
Global SAPS score	2.0 (1.9)
ACE III score	82.5 (9.2)
Faces Test score	13.8 (3.0)
RMET score	19.1 (4.2)
PSP score	61.0 (16.9)

SANS: Scale for the Assessment of Negative Symptoms; SAPS: Scale for the Assessment of Positive Symptoms; ACE III: Addenbrooke’s Cognitive Examination III; RMET: Reading the Mind in the Eyes test; PSP: Personal and Social Performance scale.

**Table 2.** Partial correlation matrix between 10 network analysis variables.

Variables	PSP	Blunted affect	Alogia	Avolition	Anhedonia	Asociality	SAPS	ACE III	Faces Test
Blunted affect	−0.26	-							
Alogia	0.06	0.33	-						
Avolition	−0.59	0.04	0.12	-					
Anhedonia	0.18	0.06	0.27	0.44	-				
Asociality	−0.5	-0.11	0.16	−0.11	0.36	-			
SAPS	−0.17	−0.12	−0.2	−0.06	−0.14	0.24	-		
ACE III	0.15	0.06	−0.15	−0.11	0.18	0.02	−0.09	-	
Faces test	0.15	−0.08	−0.1	0.13	−0.18	0.1	−0.09	0.16	-
RMET	0.16	−0.02	−0.05	0.22	−0.04	0.07	0.16	0.37	0.33

SAPS: Scale for the Assessment of Positive Symptoms; ACE III: Addenbrooke’s Cognitive Examination III; RMET: Reading the Mind in the Eyes test; PSP: Personal and Social Performance scale.

**Figure 1.**
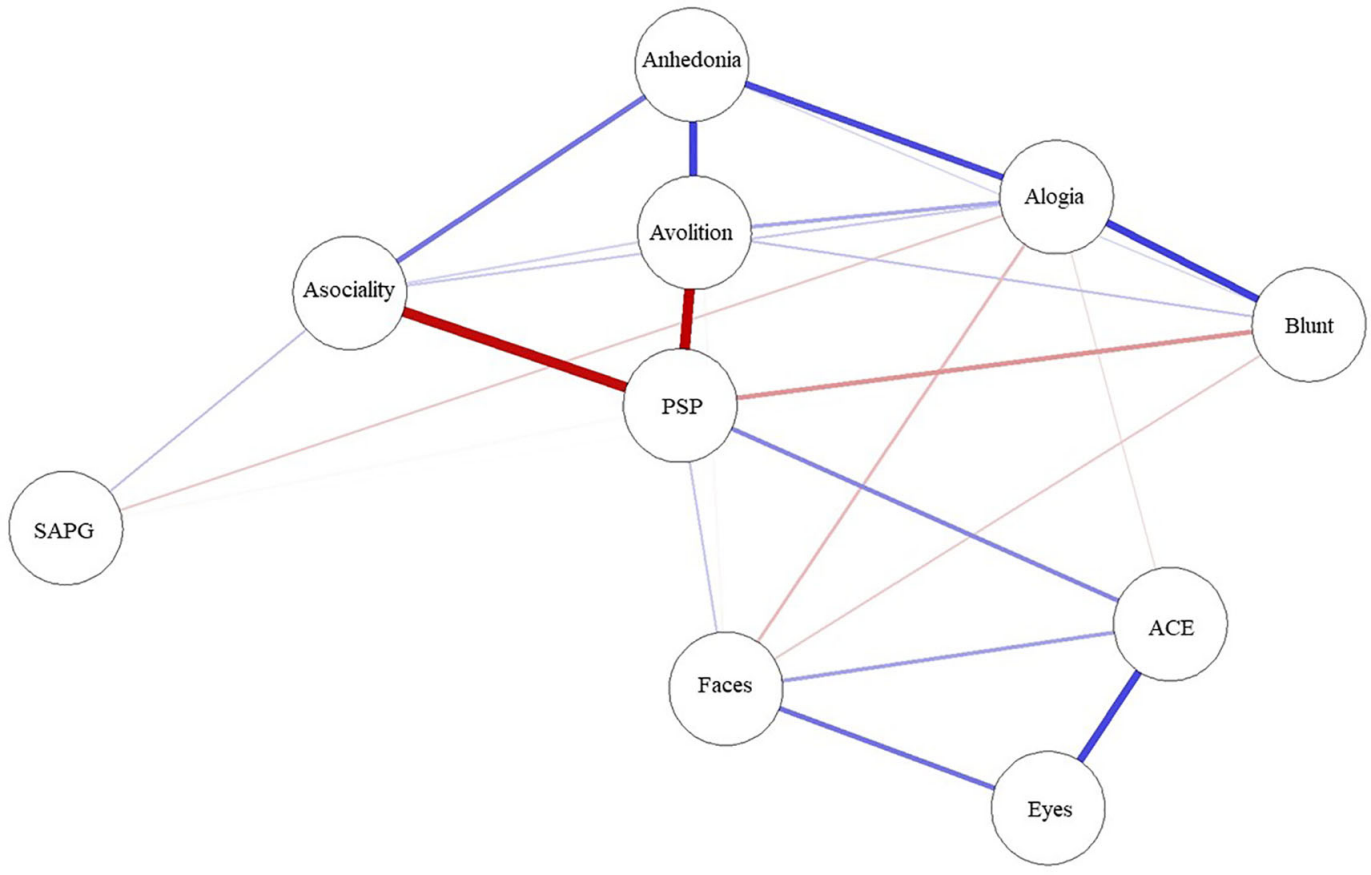
Network analysis of functioning, symptoms, neurocognition and social cognition scores. ACE: Addenbrooke’s Cognitive Examination III; Blunt: blunted affect; Eyes: Reading the Mind in the Eyes test; Faces: Faces test; PSP: Personal and Social Performance scale; SAPG: global Scale for the Assessment of Positive Symptoms score.

**Figure 2.**
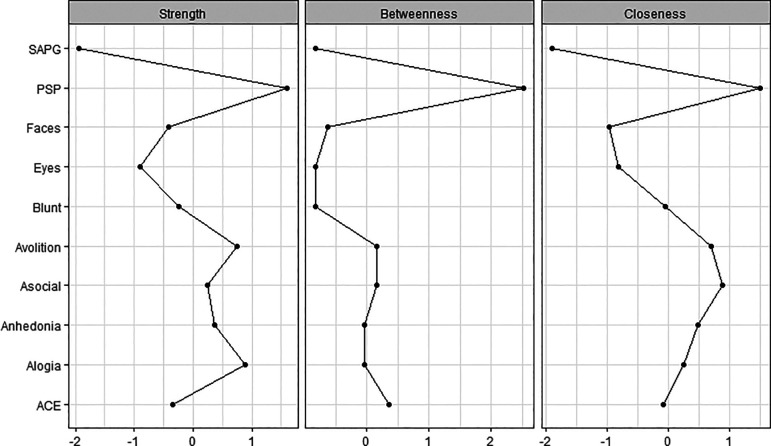
Centrality plots of functioning, symptoms, neurocognition and social cognition scores. ACE: Addenbrooke’s Cognitive Examination III; Asocial: asociality; Blunt: blunted affect; Eyes: Reading the Mind in the Eyes test; Faces: Faces test; PSP: Personal and Social Performance scale; SAPG: global Scale for the Assessment of Positive Symptoms score.

The network structure and centrality measures are demonstrated in Figures 1 and 2. The negative symptoms and cognitive function (neurocognition and social cognition) variables seem to be as two separate communities on upper and lower side of the network. The positive symptoms node appears as an isolate and farthest node from the central.

The functioning was found to be the most central node and is displayed in the center of the network structure. It connected and demonstrated inverse correlations to three subdomains of negative symptoms (asocial, avolition and blunt affect) and positive correlations to emotion recognition and neurocognition performance.

In the social cognition domain, only emotion recognition was directly connected to functioning, whereas theory of mind was connected to emotion recognition and neurocognition but not directly connected to functioning. Among cognitive function variables, neurocognition had the highest centrality index and was the strongest connection to functioning.

Among negative symptoms variables, the highest centrality index variables were asociality and avolition subdomains. Except for blunt affect, the other four negative symptoms nodes were interconnected to all other negative symptoms’ nodes.

Between negative symptoms and cognitive function, only alogia and blunt affect showed association with emotion recognition and neurocognition, whereas asociality, avolition and anhedonia showed no direct association to cognitive function.

**Figure 3.**
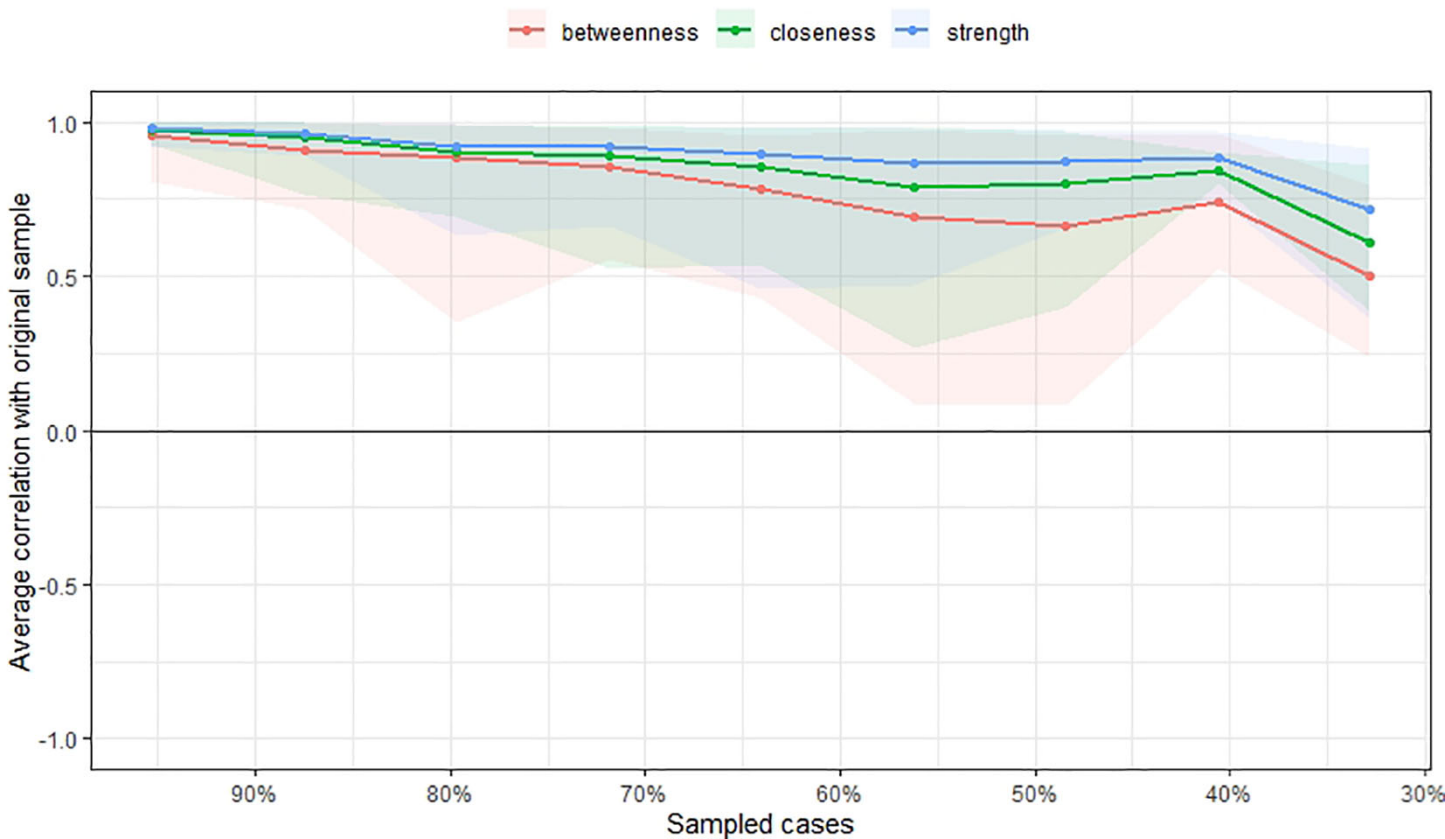
Stability of central indices.

**Figure 4.**
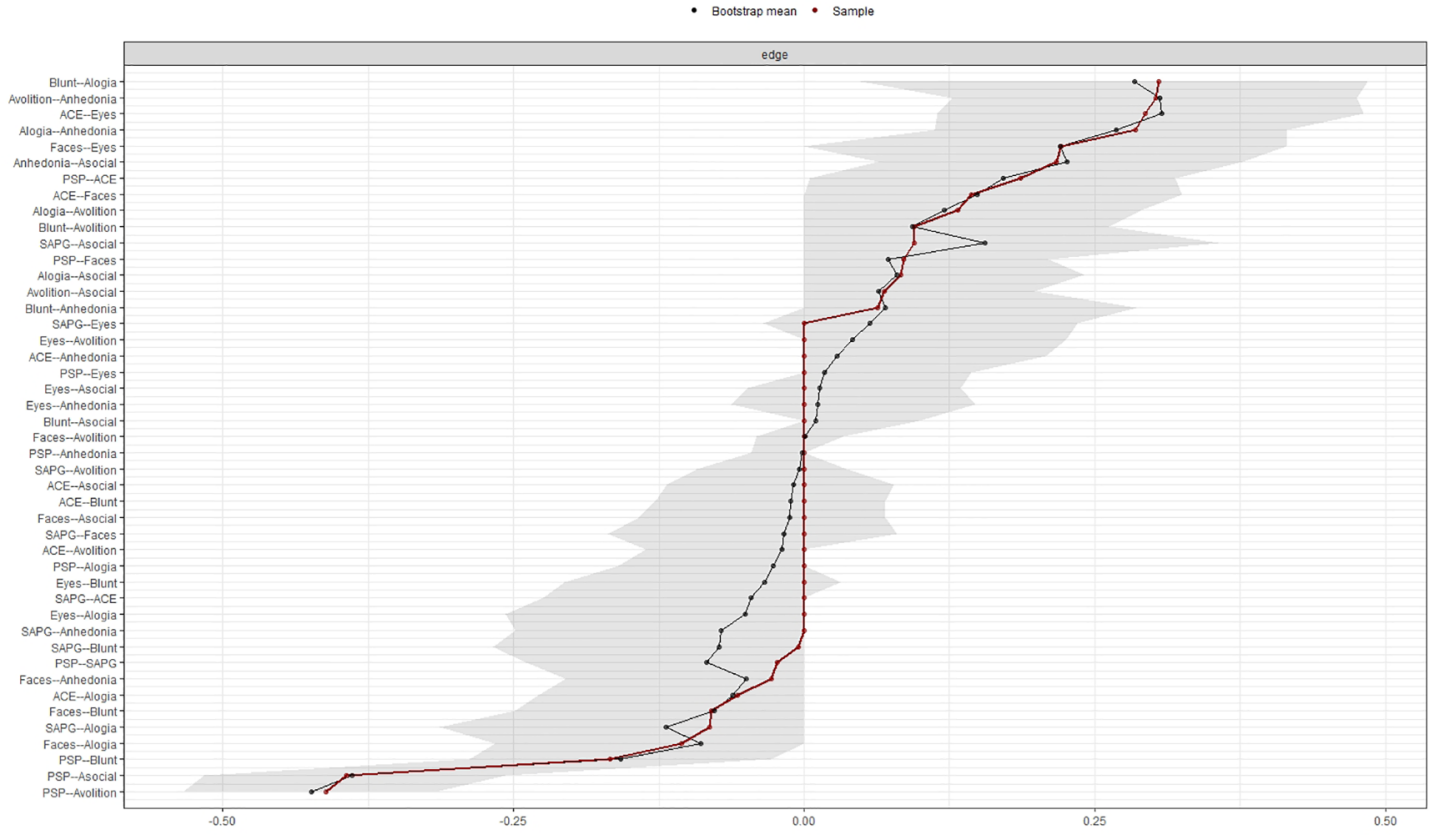
Accuracy of the edge weight estimates.

Figures 3 and 4 show the stability of central indices and accuracy of the estimated network. In brief, the CS-coefficient indicates that strength had the highest and acceptable stability (CS-coefficient 0.28); whereas closeness (CS-coefficient 0.13) and betweenness (CS-coefficient 0.2) are not stable under sub-setting cases.

## Discussion

This study used a network analysis technique to explore the complex relationships among positive symptoms, negative symptoms, neurocognition, social cognition and real-life functioning. To our knowledge, there are few studies examining this association with network approach. ^29^^-^^31^

Our results illustrate that real-life functioning is the most central and key role in the network. It has the highest centrality index in strength, betweenness and closeness. It is also connected to negative symptoms, neurocognition, and social cognition. This finding suggests that real-life functioning should be one of the main targets of schizophrenia treatment and research. Rehabilitation for patients with schizophrenia should be provided and should focus on training of everyday real-life skills. ^32^ Besides functioning, avolition and asociality appear to be the second most central and important symptoms. These two negative symptoms also firmly correlate to functioning and connect to other subdomains of negative symptoms. These results confirm the previous findings that, generally, negative symptoms and cognitive symptoms are the strongest predictors of functional outcome in schizophrenia. ^4^^,^^33^

The network also shows that neurocognition and emotion recognition are interconnected and close to real-life functioning. Therefore, neurocognition and emotion recognition training could be implemented and would benefit patients with schizophrenia. It is interesting that emotion recognition might directly improve functioning or could have an indirect effect by decreasing blunt affect symptoms. This association is in line with previous studies that blunted affect seems to be closely related to emotion recognition. ^7^^,^^34^

Conforming to previous studies, our network analysis confirms that negative and cognitive symptoms are the most important and influential on functioning in clinically stable patients with schizophrenia. ^31^^,^^35^ The result underlines the importance of evaluating and treating negative symptoms and cognitive symptoms, as positive symptoms alone have only minimal effect on real-life function. Furthermore, medications that specifically target negative symptoms, social cognition, or neurocognitive symptoms are urgently needed for patients with schizophrenia.

Regarding cognitive function and in line with previous studies on schizophrenia, this study shows that social cognition is highly correlated with neurocognition. ^7^^,^^8^ The social cognition node that directly links to functioning is emotion recognition. This connection highlights the important role of emotion recognition in patients’ real-life functioning. The result supports previous studies that found social cognition to be a strong prediction of function outcome in patients with schizophrenia. ^4^^,^^36^ On the other hand, though, theory of mind is strongly correlated to neurocognition and emotion recognition; it is not directly connected to functioning and is the most distant cognitive function node.

### Strengths and limitations

The strength of this study is that we used network analysis to examine the complex relationship among real-life functioning, clinical symptoms and cognitive functions. In addition, we also used five dimensions of negative symptoms instead of a unidimensional approach in order to avoid losing information relevant to connections with other factors and the ability of each subdomain to predict functioning. ^37^

Our study has some limitations. First, the sample size was relatively modest. Therefore, positive symptoms and neurocognition were constructed as single-measured variables to minimize the number of parameters. Second, closeness and betweenness should be interpreted with caution since their estimates showed less stability. Therefore, a larger sample might be needed to ensure a more stable estimated network. Third, on account of the inclusion of only clinically stable patients with schizophrenia, most had none or very few positive symptoms, therefore, the results might not be able to be generalized to patients with actively psychotic episodes. Lastly, the cross-sectional nature of the data could not allow proper testing of the direction of the causes and effects. Longitudinal study with long term follow-up period would be more appropriate for evaluating the effects of predictors to future functional outcome.

## Data availability

There are restrictions on publicly sharing the dataset, because of the confidential nature of the data and the informed consent given by the study participants, which has been approved by the Human Research Ethics Committee, Faculty of Medicine, Thammasat University. However, thenetwork analysis dataset (ten variables without demographic data) may be requested by contacting the corresponding author (TC) and access will be granted to researchers affiliated with an accredited institution, and reviewers. Full name, title, institution, and purpose for using the dataset should be included in an email.

## References

[1] Charernboon T : Negative and neutral valences of affective theory of mind are more impaired than positive valence in clinically stable schizophrenia patients. *Psychiatry Investig.* 2020;17(5):460–464. 10.30773/pi.2020.0040 32403211 PMC7265030

[2] Savla GN Vella L Armstrong CC : Deficits in domains of social cognition in schizophrenia: a meta-analysis of the empirical evidence. *Schizophr. Bull.* 2013;39(5):979–992. 10.1093/schbul/sbs080 22949733 PMC3756768

[3] Haro JM Novick D Bertsch J : Cross-national clinical and functional remission rates: Worldwide Schizophrenia Outpatient Health Outcomes (W-SOHO) study. *Br. J. Psychiatry.* 2011;199(3):194–201. 10.1192/bjp.bp.110.082065 21881098

[4] Fett AK Viechtbauer W Dominguez MD : The relationship between neurocognition and social cognition with functional outcomes in schizophrenia: a meta-analysis. *Neurosci. Biobehav. Rev.* 2011;35(3):573–588. 10.1016/j.neubiorev.2010.07.001 20620163

[5] Galderisi S Bucci P Mucci A : Categorical and dimensional approaches to negative symptoms of schizophrenia: focus on long-term stability and functional outcome. *Schizophr. Res.* 2013;147(1):157–162. 10.1016/j.schres.2013.03.020 23608244

[6] Green MF Kern RS Braff DL : Neurocognitive deficits and functional outcome in schizophrenia: are we measuring the “right stuff”? *Schizophr. Bull* .26:119–136. 10.1093/oxfordjournals.schbul.a033430 10755673

[7] Charernboon T : Different subdomains of negative symptoms in clinically stable patients with schizophrenia: Determining the nature of their relationships with emotion recognition, theory of mind and neurocognition. *Cogent Psychology.* 2020;7(1):1849892. 10.1080/23311908.2020.1849892

[8] Sergi MJ Rassovsky Y Widmark C : Social cognition in schizophrenia: relationships with neurocognition and negative symptoms. *Schizophr. Res.* 2007;90(1-3):316–324. 10.1016/j.schres.2006.09.028 17141477

[9] Charernboon T Patumanond J : Social cognition in schizophrenia. *Ment. Illn.* 2017;9(1):7054. 10.4081/mi.2017.7054 28479973 PMC5379217

[10] Lam BY Raine A Lee TM : The relationship between neurocognition and symptomatology in people with schizophrenia: social cognition as the mediator. *BMC Psychiatry.* 2014;14:138. 10.1186/1471-244X-14-138 24885177 PMC4026589

[11] Borsboom D Cramer AO : Network analysis: an integrative approach to the structure of psychopathology. *Annu. Rev. Clin. Psychol.* 2013;9:91–121. 10.1146/annurev-clinpsy-050212-185608 23537483

[12] American Psychiatric Association: *Diagnostic and Statistical Manual of Mental Disorders.* Washington DC: American Psychiatric Publishing;2013. 10.1176/appi.books.9780890425596

[13] Andreasen NC : *Scale for the Assessment of Positive Symptoms (SAPS).* University of Iowa Iowa City;1984.

[14] Charernboon T : Preliminary study of the Thai-version of the Scale for the Assessment of Positive Symptoms (SAPS-Thai): content validity, known-group validity, and internal consistency reliability. *Arch. Clin. Psychiatry (São Paulo).* 2019;46(1):5–8. 10.1590/0101-60830000000183

[15] Andreasen NC : *Scale for the Assessment of Negative Symptoms (SANS): Department of Psychiatry.* College of Medicine, The University of Iowa;1984.

[16] Charernboon T : Preliminary Study of the Thai Version of the Scale for the Assessment of Negative Symptoms (SANS-Thai). *Global J. Health Sci.* 2019;11(6):19. 10.5539/gjhs.v11n6p19

[17] Kirkpatrick B Fenton WS Carpenter WT Jr : The NIMH-MATRICS consensus statement on negative symptoms. *Schizophr. Bull.* 2006;32(2):214–219. 10.1093/schbul/sbj053 16481659 PMC2632223

[18] Strauss GP Ahmed AO Young JW : Reconsidering the latent structure of negative symptoms in schizophrenia: A review of evidence supporting the 5 consensus domains. *Schizophr. Bull.* 2019;45(4):725–729. 10.1093/schbul/sby169 30541136 PMC6581128

[19] Couture SM Penn DL Roberts DL . The functional significance of social cognition in schizophrenia: a review. *Schizophr. Bull.* 2006;32(Suppl1):S44–S63. 10.1093/schbul/sbl029 16916889 PMC2632537

[20] Charernboon T Jaisin K Lerthattasilp T : The Thai version of the Addenbrooke's Cognitive Examination III. *Psychiatry Investig.* 2016;13(5):571–573. 10.4306/pi.2016.13.5.571 27757137 PMC5067353

[21] Hsieh S Schubert S Hoon C : Validation of the Addenbrooke's Cognitive Examination III in frontotemporal dementia and Alzheimer's disease. *Dement. Geriatr. Cogn. Disord.* 2013;36(3-4):242–250. 10.1159/000351671 23949210

[22] Charernboon T Chompookard P : Detecting cognitive impairment in patients with schizophrenia with the Addenbrooke's Cognitive Examination. *Asian J. Psychiatr.* 2019;40:19–22. 10.1016/j.ajp.2019.01.006 30690276

[23] Charernboon T : Validity and reliability of the Thai version of the Faces Test. *J. Med. Assoc. Thail.* 2017;100(6):42–45.

[24] Baron-Cohen S Wheelwright S Hill J : The “Reading the Mind in the Eyes” Test revised version: a study with normal adults, and adults with Asperger syndrome or high-functioning autism. *J. Child Psychol. Psychiatry.* 2001;42(2):241–251. 10.1111/1469-7610.00715 11280420

[25] Charernboon T Lerthattasilp T : The Reading the Mind in the Eyes Test: Validity and reliability of the Thai version. *Cogn. Behav. Neurol.* 2017;30(3):98–101. 10.1097/WNN.0000000000000130 28926417

[26] Morosini PL Magliano L Brambilla L : Development, reliability and acceptability of a new version of the DSM-IV Social and Occupational Functioning Assessment Scale (SOFAS) to assess routine social functioning. *Acta Psychiatr. Scand.* 2000;101(4):323–329. 10.1111/j.1600-0447.2000.tb10933.x 10782554

[27] Srisurapanont M Arunpongpaisal S Chuntaruchikapong S : Cross-cultural validation and inter-rater reliability of the Personal and Social Performance scale, Thai version. *J. Med. Assoc. Thai.* 2008;91(10):1603–1608. 18972906

[28] Epskamp S Borsboom D Fried EI : Estimating psychological networks and their accuracy: A tutorial paper. *Behav. Res.* 2018;50:195–212. 10.3758/s13428-017-0862-1 28342071 PMC5809547

[29] Hajdúk M Penn DL Harvey PD : Social cognition, neurocognition, symptomatology, functional competences and outcomes in people with schizophrenia - A network analysis perspective. *J. Psychiatr. Res.* 2021;144:8–13. 10.1016/j.jpsychires.2021.09.041 34592511 PMC8665006

[30] Lui SSY Zhang RT Lau WYS : Prospective memory influences social functioning in people with first-episode schizophrenia: A network analysis and longitudinal study. *J. Clin. Psychiatry.* 2022;83(2):21m14114. 10.4088/JCP.21m14114 35143123

[31] Galderisi S Rucci P Kirkpatrick B : Interplay among psychopathologic variables, personal resources, context-related factors, and real-life functioning in individuals with schizophrenia: A network analysis. *JAMA Psychiat.* 2018;75(4):396–404. 10.1001/jamapsychiatry.2017.4607 29450447 PMC5875306

[32] Farkas M : The vision of recovery today: what it is and what it means for services. *World Psychiatry.* 2007;6(2):68–74. 18235855 PMC2219905

[33] Chue P Lalonde JK : Addressing the unmet needs of patients with persistent negative symptoms of schizophrenia: emerging pharmacological treatment options. *Neuropsychiatr. Dis. Treat.* 2014;10:777–789. 10.2147/NDT.S43404 24855363 PMC4020880

[34] Ditlevsen JV Simonsen A Bliksted VF : Predicting mentalizing deficits in first-episode schizophrenia from different subdomains of negative symptoms. *Schizophr. Res.* 2020;215:439–441. 10.1016/j.schres.2019.10.036 31672383

[35] Ventura J Hellemann GS Thames AD : Symptoms as mediators of the relationship between neurocognition and functional outcome in schizophrenia: a meta-analysis. *Schizophr. Res.* 2009;113(2-3):189–199. 10.1016/j.schres.2009.03.035 19628375 PMC2825750

[36] Irani F Seligman S Kamath V : A meta-analysis of emotion perception and functional outcomes in schizophrenia. *Schizophr. Res.* 2012;137(1-3):203–211. 10.1016/j.schres.2012.01.023 22341200 PMC3351501

[37] Marder SR Galderisi S : The current conceptualization of negative symptoms in schizophrenia. *World Psychiatry.* 2017;16(1):14–24. 10.1002/wps.20385 28127915 PMC5269507

