# Supplementary material for: Interplay among positive and negative symptoms, neurocognition, social cognition, and functioning in clinically stable patients with schizophrenia: a network analysis
Source: F1000Res. 2022 Apr 13;10:1258. Originally published 2021 Dec 8. [Version 3] doi: 10.12688/f1000research.74385.3 (PMC9021676; doi:10.12688/f1000research.74385.3)
Supplement: Peer review history

# Peer review: Reviewer response for version 3

**DOI:** 10.5256/f1000research.125399.r134744

**Published:** 2022-04-20

**Authors:** Haruo Fujino

**Affiliations:** 1 United Graduate School of Child Development, Osaka University, Osaka, Japan

Thank you for your revision. The revisions improved the clarity of the section. Further discussion would be discretionary, not mandatory.

Is the work clearly and accurately presented and does it cite the current literature?

Partly

If applicable, is the statistical analysis and its interpretation appropriate?

Partly

Are all the source data underlying the results available to ensure full reproducibility?

Partly

Is the study design appropriate and is the work technically sound?

Partly

Are the conclusions drawn adequately supported by the results?

Partly

Are sufficient details of methods and analysis provided to allow replication by others?

Partly

Reviewer Expertise:

Research on cognitive and social functioning in schizophrenia

I confirm that I have read this submission and believe that I have an appropriate level of expertise to confirm that it is of an acceptable scientific standard.

# Peer review: Reviewer response for version 2

**DOI:** 10.5256/f1000research.122220.r127161

**Published:** 2022-03-28

**Authors:** Pichai Ittasakul

**Affiliations:** 1 Department of Psychiatry, Faculty of Medicine, Ramathibodi Hospital, Mahidol University, Bangkok, Thailand

No further comments to make.

Is the work clearly and accurately presented and does it cite the current literature?

Yes

If applicable, is the statistical analysis and its interpretation appropriate?

Yes

Are all the source data underlying the results available to ensure full reproducibility?

Yes

Is the study design appropriate and is the work technically sound?

Yes

Are the conclusions drawn adequately supported by the results?

Yes

Are sufficient details of methods and analysis provided to allow replication by others?

Yes

Reviewer Expertise:

Electroconvulsive therapy, schizophrenia, mood disorders

I confirm that I have read this submission and believe that I have an appropriate level of expertise to confirm that it is of an acceptable scientific standard.

# Peer review: Reviewer response for version 2

**DOI:** 10.5256/f1000research.122220.r127162

**Published:** 2022-03-24

**Authors:** Haruo Fujino

**Affiliations:** 1 United Graduate School of Child Development, Osaka University, Osaka, Japan

Thank you for the author's effort to revise the manuscript. Now, the strengths and limitations of the approach were clearly stated. 

 Only a minor issue requires revision in the Discussion section. In the Discussion section, please discuss the similarities and differences between current and previous findings (ref: 29-31), as this reviewer described in the first round review. It would enhance the significance of this paper.

Is the work clearly and accurately presented and does it cite the current literature?

Partly

If applicable, is the statistical analysis and its interpretation appropriate?

Partly

Are all the source data underlying the results available to ensure full reproducibility?

Partly

Is the study design appropriate and is the work technically sound?

Partly

Are the conclusions drawn adequately supported by the results?

Partly

Are sufficient details of methods and analysis provided to allow replication by others?

Partly

Reviewer Expertise:

Research on cognitive and social functioning in schizophrenia

I confirm that I have read this submission and believe that I have an appropriate level of expertise to confirm that it is of an acceptable scientific standard, however I have significant reservations, as outlined above.

# Response

**Published:** 2022-04-06

**Authors:** Thammanard Charernboon

**Affiliations:** Thammasat University, Thailand

We appreciate the time and efforts by the editor and reviewers in reviewing this manuscript. We have addressed all issues indicated in the review report and believed that the revised version can meet the journal publication requirements

 Reviewer 2:

 Thank you for the author's effort to revise the manuscript. Now, the strengths and limitations of the approach were clearly stated. Only a minor issue requires revision in the Discussion section. In the Discussion section, please discuss the similarities and differences between current and previous findings (ref: 29-31), as this reviewer described in the first round review. It would enhance the significance of this paper.

 Ans: We’ve added more discussion regarding the comparison with previous network analysis findings as follows: 

*"Compared with the existing network analysis studies on determinants of functioning in schizophrenia, overall, our results are consistent with other studies. 29, 31 First, these studies support that functioning was found to be the most central node, whereas positive symptoms appeared as the farthest node. Second, our results confirm that negative symptoms, neurocognition and social cognition are important contributors to functioning, and better understood as a separate construct. 29, 31 Also consistent with Hajduk et al., the RMET was strongly connected to emotion recognition but not directly connected to functioning. 31 However, comparing details of the networks between these studies should be interpreted with caution. Each study used significantly different measurements and variables. For example, each study used different functional assessment scales; and our study used five dimensions of negative symptoms, while the others used two and unidimensional approaches. 29, 31"*

# Peer review: Reviewer response for version 1

**DOI:** 10.5256/f1000research.78132.r122301

**Published:** 2022-02-21

**Authors:** Haruo Fujino

**Affiliations:** 1 United Graduate School of Child Development, Osaka University, Osaka, Japan

The paper “Interplay among positive and negative symptoms, neurocognition, social cognition, and functional outcome in clinically stable patients with schizophrenia: a network analysis” focuses on the relationships among psychiatric symptoms, cognitive function, and social functioning in patients with clinically stable schizophrenia using network analysis. The application of the analysis in these data is interesting and would have potential contributions to the literature.

 I have the following comments for the first version of the paper. I hope that these comments could help improve the reporting of the paper.

**Major comments:**

- The variable selection is a critically important factor that characterizes the correlation network. Please include the justification for the selected variables, particularly negative symptoms, in this study. The explanation would facilitate understanding of the focus and context of the study.
- Information about the stability and accuracy of the network should be included in the Methods and Results sections. The information would provide useful information for interpreting the strength and instability of the network.
- The sentence in the Discussion section, "To our knowledge, there are few studies examining this association with network approach." does not provide information or citations. I recommend including recent studies using the network approach (as shown in the references below.) The inclusion of the recent papers and discussion on the current and previous findings would enhance the significance and clarify the nature of the research:
  - Hajdúk M, Penn DL, Harvey PD, Pinkham AE. Social cognition, neurocognition, symptomatology, functional competences and outcomes in people with schizophrenia - A network analysis perspective ^1^.
  - Pena-Garijo J, Monfort-Escrig C. The centrality of secure attachment within an interacting network of symptoms, cognition, and attachment dimensions in persons with schizophrenia-spectrum disorders: A preliminary study ^2^.
  - Lui SSY, Zhang RT, Lau WYS, *et al.* Prospective Memory Influences Social Functioning in People With First-Episode Schizophrenia: A Network Analysis and Longitudinal Study ^3^.
- In the Discussion section, the sentence “ *Our study also supports a multidimensional model of negative symptoms…*”, was not supported by the results. In the Results section, the sentence “ *The negative symptoms and cognitive function variables seem to be as two separate communities…*”, suggested that negative symptoms constructed a cluster. Please remove this part.
- Regarding the strengths and limitations, please discuss how sample size limits the validity of the study or leads to potential bias, including the results of network stability and accuracy.

**Minor comments:**

- Please revise the word “functional outcome” in the title because the outcome implies a longitudinal outcome.
- In the Discussion, the sentence “ *The finding supports the notion that real-life function should serve as the main target of treatment…*”, needs revision because the network analysis only showed the cross-sectional associations between the variables. A more careful statement is preferable.
- The sentence “ *positive symptoms are less important and less influential on functioning in clinically stable…*” requires revision. Although this is the reviewer’s opinion, positive symptoms are clinically important targets in schizophrenia. It may have less influence on social functioning in groups. However, it is an essential treatment target in clinical practice.

Is the work clearly and accurately presented and does it cite the current literature?

Partly

If applicable, is the statistical analysis and its interpretation appropriate?

Partly

Are all the source data underlying the results available to ensure full reproducibility?

Partly

Is the study design appropriate and is the work technically sound?

Partly

Are the conclusions drawn adequately supported by the results?

Partly

Are sufficient details of methods and analysis provided to allow replication by others?

Partly

Reviewer Expertise:

Research on cognitive and social functioning in schizophrenia

I confirm that I have read this submission and believe that I have an appropriate level of expertise to confirm that it is of an acceptable scientific standard, however I have significant reservations, as outlined above.

## References

[1] : Social cognition, neurocognition, symptomatology, functional competences and outcomes in people with schizophrenia - A network analysis perspective. *J Psychiatr Res* .2021;144: 10.1016/j.jpsychires.2021.09.041 8-13 10.1016/j.jpsychires.2021.09.041 34592511PMC8665006

[2] : The centrality of secure attachment within an interacting network of symptoms, cognition, and attachment dimensions in persons with schizophrenia-spectrum disorders: A preliminary study. *J Psychiatr Res* .135: 10.1016/j.jpsychires.2021.01.002 60-67 10.1016/j.jpsychires.2021.01.002 33450466

[3] : Prospective Memory Influences Social Functioning in People With First-Episode Schizophrenia: A Network Analysis and Longitudinal Study. *J Clin Psychiatry* .2022;83(2) : 10.4088/JCP.21m14114 10.4088/JCP.21m14114 35143123

# Response

**Published:** 2022-03-07

**Authors:** Thammanard Charernboon

**Affiliations:** Thammasat University, Thailand

We appreciate the time and efforts by the editor and reviewers in reviewing this manuscript. We have addressed all issues indicated in the review report, and believed that the revised version can meet the journal publication requirements.

**Reviewer 2**

- **Major comments:** The variable selection is a critically important factor that characterizes the correlation network. Please include the justification for the selected variables, particularly negative symptoms, in this study. The explanation would facilitate understanding of the focus and context of the study.

*We greatly appreciate the reviewer’s efforts to carefully review the paper and the valuable suggestions offered. As suggested, we have added more explanations in the introduction and methods sections as follows:*

***Introduction***

*“… While all of the symptoms are believed to affect functional outcome to some extent, negative symptoms, neurocognitive, and social cognition seem to have the highest impact on functioning. ^4^ For example, previous studies generally demonstrate that negative symptoms have associations with poorer levels of functioning. ^5^ Neurocognitive impairment is a predictor for low levels of a real-world functioning. ^6^ Moreover, in the last decade, social cognition, a relatively new concept, appears to be a strong predictor of social abilities and real-world function.  Theory of mind and emotion recognition are the most extensively studied domains in social cognition study, and impairment in these tasks are risk factors for poor social and functional outcome in patients with schizophrenia.” ^4,6^*

***Methods***

*“… In this study, negative symptoms were classified into five subdomains i.e., blunted affect, alogia, anhedonia, avolition, and asociality as suggested by the NIMH-MATRICS consensus statement on negative symptoms. ^17^ Each subdomain has a maximum score of five, which is computed from the average scores of the relevant items of each subdomain. It should be noted that inattention subdomain was not included in the analysis because of its overlap with neurocognitive assessment.*

*We used five dimensions of negative symptoms instead of a unidimensional approach because several recent factor analysis studies suggest that a unidimensional model of negative symptoms does not adequately capture the complexity of negative symptoms. ^17, 18^ Furthermore, previous study showed that negative symptoms subdomains seem to have a different relationship with functioning. For example, avolition and apathy seem to be a stronger predictor of poor outcomes than blunted affect ^4, 19^. Therefore, utilizing a multidimensional model would avoid losing information relevant to the connection with other variables and the ability of each subdomain to predict functioning.”*

  

- Information about the stability and accuracy of the network should be included in the Methods and Results sections. The information would provide useful information for interpreting the strength and instability of the network.

*We have added the information about the stability and accuracy of the network as follows:*

***Statistical analysis***

*“The accuracy and stability of the centrality indices of the estimated network were examined using edge weight accuracy and case-dropping bootstrap method on the 500 samples. We utilized recommended cut-off for Coefficient Stability (CS-coefficient, correlation = 0.7) at least 0.25 for considering stable network.” ^28^*

***Results***

*“Figures 3 and 4 show the stability of central indices and accuracy of the estimated network. In brief, the CS-coefficient indicates that strength had the highest and acceptable stability (CS-coefficient 0.28); whereas closeness (CS-coefficient 0.13) and betweenness (CS-coefficient 0.2) are not stable under sub-setting cases.”*

*Please see Fig 3 (Stability of central indices) and Fig 4 (Accuracy of the edge weight estimates) in the revised manuscript.*

***Limitations***

*“… Second, closeness and betweenness should be interpreted with caution since their estimates showed less stability. Therefore, a larger sample might be needed to ensure a more stable estimated network.”*

  

- The sentence in the Discussion section, "To our knowledge, there are few studies examining this association with network approach." does not provide information or citations. I recommend including recent studies using the network approach (as shown in the references below.) The inclusion of the recent papers and discussion on the current and previous findings would enhance the significance and clarify the nature of the research:
- Hajdúk M, Penn DL, Harvey PD, Pinkham AE. Social cognition, neurocognition, symptomatology, functional competences and outcomes in people with schizophrenia - A network analysis perspective1.
- Pena-Garijo J, Monfort-Escrig C. The centrality of secure attachment within an interacting network of symptoms, cognition, and attachment dimensions in persons with schizophrenia-spectrum disorders: A preliminary study2.
- Lui SSY, Zhang RT, Lau WYS, et al. Prospective Memory Influences Social Functioning in People With First-Episode Schizophrenia: A Network Analysis and Longitudinal Study3.

*I have added some references to this sentence as follows:*

*"29    Hajdúk M, Penn DL, Harvey PD, et al.: Social cognition, neurocognition, symptomatology, functional competences and outcomes in people with schizophrenia - A network analysis perspective. J. Psychiatr. Res. 2021;144:8-13. 10.1016/j.jpsychires.2021.09.041*

*30     Lui SSY, Zhang RT, Lau WYS, et al.: Prospective memory influences social functioning in people with first-episode schizophrenia: A network analysis and longitudinal study. J. Clin. Psychiatry. 2022;83(2):21m14114.  10.4088/JCP.21m14114.*

*31    Galderisi S, Rucci P, Kirkpatrick B, et al.: Interplay among psychopathologic variables, personal resources, context-related factors, and real-life functioning in individuals with schizophrenia: A network analysis. JAMA Psychiat. 2018;75(4):396–404. 29450447 10.1001/jamapsychiatry.2017.4607"*

  

- In the Discussion section, the sentence “Our study also supports a multidimensional model of negative symptoms…”, was not supported by the results. In the Results section, the sentence “The negative symptoms and cognitive function variables seem to be as two separate communities…”, suggested that negative symptoms constructed a cluster. Please remove this part.

*These sentences have been removed as suggested.*

  

- Regarding the strengths and limitations, please discuss how sample size limits the validity of the study or leads to potential bias, including the results of network stability and accuracy.

*We have rewritten the limitations. The results of the network stability and sample sizes were also added and discussed as follows:*

*“Our study has some limitations. First, the sample size was relatively modest. Therefore, positive symptoms and neurocognition were constructed as single-measured variables to minimize the number of parameters. Second, closeness and betweenness should be interpreted with caution since their estimates showed less stability. Therefore, a larger sample might be needed to ensure a more stable estimated network. Third, on account of the inclusion of only clinically stable patients with schizophrenia, most had none or very few positive symptoms, therefore, the results might not be able to be generalized to patients with actively psychotic episodes. Lastly, the cross-sectional nature of the data could not allow proper testing of the direction of the causes and effects. Longitudinal study with long term follow-up period would be more appropriate for evaluating the effects of predictors to future functional outcome.”*

 ***********************

- **Minor comments:**
  - Please revise the word “functional outcome” in the title because the outcome implies a longitudinal outcome.

*“Functional outcome” was substituted with “functioning” in the title and other parts in the manuscript. * 

  

- In the Discussion, the sentence “The finding supports the notion that real-life function should serve as the main target of treatment…”, needs revision because the network analysis only showed the cross-sectional associations between the variables. A more careful statement is preferable.

*I have edited this statement and softened it as follows:*

*“Our results illustrate that real-life functioning is the most central and key role in the network. It has the highest centrality index in strength, betweenness and closeness. It is also connected to negative symptoms, neurocognition, and social cognition. This finding suggests that real-life functioning should be one of the main targets of schizophrenia treatment and research. Rehabilitation for patients with schizophrenia should be provided and should focus on training of everyday real-life skills.”*

  

- The sentence “positive symptoms are less important and less influential on functioning in clinically stable…” requires revision. Although this is the reviewer’s opinion, positive symptoms are clinically important targets in schizophrenia. It may have less influence on social functioning in groups. However, it is an essential treatment target in clinical practice.

*I have revised this statement to make it focuses more on negative and cognitive symptoms as follows:*

*“Conforming to previous studies, our network analysis confirms that negative and cognitive symptoms are the most important and influential on functioning in clinically stable patients with schizophrenia.” ^35, 36^*

# Peer review: Reviewer response for version 1

**DOI:** 10.5256/f1000research.78132.r102245

**Published:** 2021-12-17

**Authors:** Pichai Ittasakul

**Affiliations:** 1 Department of Psychiatry, Faculty of Medicine, Ramathibodi Hospital, Mahidol University, Bangkok, Thailand

Comments to the Author, I would like to clarify the following:

- How did the investigator obtain informed consent from the participants in this study? This should be stated in the methods.
- In the methods, the measurement (SAPS, SANS, and PSP, ACE-II, Faces, RMET were administered by psychiatrists and clinical psychologists. How is the degree of agreement (interrater reliability) among independent raters?
- The investigators should consider mentioning the limitation of this study: a cross-sectional nature of the study does not allow appropriate testing of the direction of the effect.

Is the work clearly and accurately presented and does it cite the current literature?

Yes

If applicable, is the statistical analysis and its interpretation appropriate?

Yes

Are all the source data underlying the results available to ensure full reproducibility?

Yes

Is the study design appropriate and is the work technically sound?

Yes

Are the conclusions drawn adequately supported by the results?

Yes

Are sufficient details of methods and analysis provided to allow replication by others?

Yes

Reviewer Expertise:

Electroconvulsive therapy, schizophrenia, mood disorders

I confirm that I have read this submission and believe that I have an appropriate level of expertise to confirm that it is of an acceptable scientific standard, however I have significant reservations, as outlined above.

# Response

**Published:** 2022-03-07

**Authors:** Thammanard Charernboon

**Affiliations:** Thammasat University, Thailand

We appreciate the time and efforts of the editor and reviewers in reviewing this manuscript. We have addressed all issues indicated in the review report and believed that the revised version can meet the journal publication requirements.

**Reviewer 1**

- How did the investigator obtain informed consent from the participants in this study? This should be stated in the methods.

We have added the suggested information as follows:

 “The participants were invited to participate in this study by our research assistants during outpatient visits. The study was approved by the Human Research Ethics Committee of Thammasat University (No. MTU-EC-PS-0-191/60). Written informed consent forms were obtained from all participants. Participants had a right to withdraw from the study at any time.”

  

- In the methods, the measurement (SAPS, SANS, and PSP, ACE-III, Faces, RMET were administered by psychiatrists and clinical psychologists. How is the degree of agreement (interrater reliability) among independent raters?

We did not evaluate inter-rater reliabilities in our study. However, a previous study has explored the interrater reliability of the Thai version of the ACE-III. [Charernboon T, Jaisin K, Lerthattasilp T. The Thai version of the Addenbrooke’s Cognitive Examination III. Psychiatry Investigation, 2016; 13: 571-573.] A Thai version of the ACE-III had an excellent inter-rater reliability with a Pearson’s correlation of 1.0 (p < 0.001).

 Srisurapanont *et al.* also evaluate the inter-rater reliability of the PSP, Thai version. The intraclass correlation coefficients of the PSP total score was 0.75 (p<0.001). [Srisurapanont M, et al. Cross-cultural validation and inter-rater reliability of the Personal and Social Performance Scale, Thai version. J Med Assc Thai 2008; 91: 1603-8.]. These properties have been added to the manuscript.

 On the other hand, the Faces and RMET are self-rated, so inter-rater reliabilities are not available.

  

- The investigators should consider mentioning the limitation of this study: a cross-sectional nature of the study does not allow appropriate testing of the direction of the effect.

Thank you for the recommendation. We have added this limitation as follows:

 “... Lastly, the cross-sectional nature of the data could not allow proper testing of the direction of the causes and effects. A longitudinal study with long term follow-up period would be more appropriate for evaluating the effects of predictors to future functional outcome.”
